# Rapid Detection of Clenbuterol Residues in Pork Using Enhanced Raman Spectroscopy

**DOI:** 10.3390/bios12100859

**Published:** 2022-10-11

**Authors:** Qinghui Guo, Yankun Peng, Xinlong Zhao, Yahui Chen

**Affiliations:** College of Engineering, National R&D Center for Agro-Processing Equipment, China Agricultural University, 17 Qinghua East Road, Haidian, Beijing 100083, China

**Keywords:** clenbuterol, meat, SERS, aggregating compounds

## Abstract

Clenbuterol (CB) is a synthetic *β*-receptor agonist which can be used to improve carcass leanness in swine, but its residues in pork also pose health risks. In this report, surface-enhanced Raman scattering (SERS) technology was used to achieve rapid detection and identification of clenbuterol hydrochloride (CB) residues. First, the effects of several different organic solvents on the extraction efficiency were compared, and it was found that clenbuterol in pork had a better enhancement effect using ethyl acetate as an extraction agent. Then, SERS signals of clenbuterol in different solvents were compared, and it was found that clenbuterol had a better enhancement effect in an aqueous solution. Therefore, water was chosen as the solvent for clenbuterol detection. Next, enhancement effect was compared using different concentration of sodium chloride solution as the aggregating compound. Finally, pork samples with different clenbuterol content (1, 3, 5, 7, 9, and 10 µg/g) were prepared for quantitative analysis. The SERS spectra of samples were collected with 0.5 mol/L of NaCl solution as aggregating compound and gold colloid as an enhanced substrate. Multiple scattering correction (MSC) and automatic Whittaker filter (AWF) were used for preprocessing, and the fluorescence background contained in the original Raman spectra was removed. A unary linear regression model was established between SERS intensity at 1472 cm^-1^ and clenbuterol content in pork samples. The model had a better linear relationship with a correlation coefficient *R*^2^ of 0.99 and a root mean square error of 0.263 µg/g. This method can be used for rapid screening of pork containing clenbuterol in the market.

## 1. Introduction

Clenbuterol (CB) is a synthetic *β*-receptor agonist and its chemical name is 4-amino-α-((tertbutyl amino) methyl)-3,4-dichlorobenzylalcohol [1]. Clenbuterol can promote animal growth and increase feeding efficiency by reducing fat deposition and enhancing protein accretion, which is used as a breeding promoter by some livestock farmers and breeding enterprises [2]. Feeding livestock with drugs such as clenbuterol can lead to deposits forming in the muscle or liver which can be difficult to break down. Humans who eat food containing CB are susceptible to such health issues as nausea, headaches, and even death [3,4,5,6]. Many countries have banned the use of CB in livestock and poultry production [7]. Therefore, it is crucial to detect and control any meat containing clenbuterol in the market.

There are many ways to detect CB, including high performance liquid chromatography (HPLC) [8], capillary electrophoresis (CE) [9], enzyme-linked immunosorbent assay (ELISA) [10,11], gas chromatography-mass spectrometry (GC-MS) [12], and liquid chromatography with tandem mass spectrometry (LC-MS) [13,14]. Although these detection methods have the advantage of high accuracy, professional operators, complex extraction steps, and expensive equipment are also needed. Enzyme-linked immunosorbent assay (ELISA) is a faster identification method but requires the preparation of antibodies, which is expensive and prone to inactivation. Therefore, accurate, fast, and efficient detection methods for clenbuterol are needed to meet the quality and safety control requirements of food. 

Surface-enhanced Raman spectroscopy (SERS) is a novel analytical tool providing ultrasensitive detection based on the enhanced local electromagnetic field near the nanostructured noble metal surfaces, which allows trace level detection in combination with detailed qualitative information, giving characteristic responses for each molecule that are true fingerprints of a certain compound [15,16]. SERS technology can enhance the Raman signal intensity of target molecules by 10^4^–10^6^ times. In recent years, SERS technology has achieved significant progress in detection for trace analysis of prohibited or restricted chemicals, such as food additives [17], pesticides [18], and antibiotics [19].

Sample preparation has always been the main bottleneck of chemical residue detection in food. In particular, Raman spectra contain strong background signals and irrelevant characteristic bands because livestock and poultry meat contain more complex substances such as protein, fat, and pigment, when target substance in livestock and poultry meat are detected using SERS. The fluorescence background and characteristic bands produced by irrelevant components will greatly disturb the Raman signals of the target substance, and even mask the Raman signal of the target substance.

The pre-processing steps of clenbuterol detection using liquid chromatography with tandem mass spectrometry (LC-MS) are as follows: extraction, centrifugation, degreasing, filtration, concentration, and purification [20]. These steps take a long time and require more reagents and drugs. In recent years, the quick, easy, cheap, effective, rugged, and safe (QuEChERS) method has been developed rapidly, which simplifies the extraction and purification process and reduces the time duration [21]. The process mainly includes extraction, magnesium sulfate dehydration, removal of the fat, and concentration. However, C_18_ sorbent, PSA sorbent, enhanced matrix removal-lipid kit, MgSO_4,_ and other reagents to remove the matrix are often used in the purification process, which is expensive and time consuming. Therefore, the development of a simple and effective sample pretreatment process can greatly improve time and efficiency.

In this report, the effects of several different organic solvents on the extraction results were explored. Then, comparing clenbuterol signals in several different solvents, ultrapure water was chosen as the solvent for clenbuterol detection. Finally, the unary linear regression model between clenbuterol content in pork and SERS intensity was established under optimal detection conditions. 

## 2. Materials and Methods

### 2.1. Reagents and Materials

Chloroauric acid tetrahydrate (HAuCl_4_∙4H_2_O) was purchased from Shenyang Jinke Reagent Co., LTD (Shenyang, China). The pure product of clenbuterol hydrochloride (C_12_H_18_Cl_2_N_2_O·HCl, 98.8%) was received from Beijing Shengda-Huijie Technology Co., LTD (Beijing, China). Sodium citrate (C_6_H_5_Na_3_O_7_) and sodium chloride were obtained from Shanghai Macklin Technology Co., LTD (Shanghai, China). Ethyl acetate (C_4_H_8_O_2_), acetone, methanol, and acetonitrile were purchased from Beijing Chemical Plant. All reagents were dissolved and diluted with ultrapure water. The fresh pig muscle tissues were bought at Beijing Xing-fu Supermarket. All glassware was cleaned with aqua regia before the test.

### 2.2. Experimental Equipment

Electronic balance (ME204) was obtained from Mettler Toledo Measurement Technology Co. Ltd. (Changzhou, China). Vortex mixing (QL-901) was received from Haimen Qilin Bell Instrument Manufacturing Co., Ltd. (Nantong, China). Constant temperature magnetic stirrer (88-1) and centrifuge (TDZ5-WS) were purchased from Changzhou Guohua Electric Co., Ltd. (Guangzhou, China) and Hunan Xiang-xi Instrument Equipment Co., Ltd. (Changsha, China), respectively.

The Raman spectra acquisition system used in this paper consists of a Raman spectrometer (ATP5020, Aopu Tiancheng Technology Co., LTD, (Xiamen, China)), a 785 nm laser, optical fiber and power. For spectra acquisition, the integral time was set to 4 s and laser power was measured to at 100 mW. A UV/vis Spectrophotometer 756S was used to collect UV/vis absorption spectra of gold colloid in the 1190–1100 nm spectral range. The TEM image of gold colloid was obtained by a Transmission electron microscopy (JEM-1200EX, JEOL Ltd., Tokyo, Japan).

### 2.3. Synthesis of Gold Colloid

**Figure 1:**
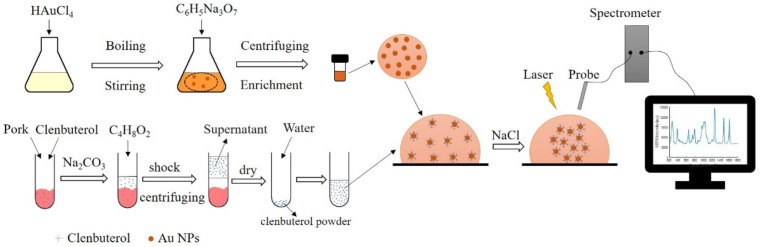
Schematic illustration of preparation and clenbuterol detection process of gold colloid.

Gold colloid was synthesized by reducing chloroauric acid tetrahydrate with sodium citrate [22]. The procedure is displayed in Figure 1. First, 1 g of solid chloroauric acid was added to 99 mL water to fully dissolve, and then diluted 100 times with water to obtain chloroauric acid solution of 0.01% by weight. Then, sodium citrate solution of 1% by weight was obtained by dissolving 0.1 g sodium citrate in 9.9 mL water. Third, 3 mL of sodium citrate solution was quickly added to the 500 mL of boiling chloroauric acid solution and boiling continued for 15 min under vigorous stirring. The solution gradually turned wine red. After cooling to room temperature, the gold colloid was centrifuged and concentrated at 3000 r/min for 20 min. The supernatant was removed, and the concentrated nanoparticles were placed in a freezer at 4 °C for future use.

### 2.4. Sample Pretreatment

A total of 10 mg of clenbuterol was added to 100 mL of ultrapure water to obtain 100 µg/mL of clenbuterol aqueous solution. The 100 µg/mL of clenbuterol aqueous solution was diluted to 6 different concentrations of 1, 3, 5, 7, 9, and 10 µg/mL by ultrapure water. Tissue samples (2 ± 0.01 g) were ground, weighed, and added to a 15 mL plastic centrifuge tube. Then, 200 µL of 10 µg/mL clenbuterol solution was added. The mixture was vortexed and mixed for 1 min. Thus, a standard sample of pork containing clenbuterol (10 µg/g) was obtained. Likewise, pork samples containing clenbuterol (1, 3, 5, 7, 9, and 10 µg/g) were prepared by adding 200 µL of clenbuterol aqueous solutions with different concentrations. The clenbuterol content of the prepared pork samples was calculated by mass ratio of the actual added clenbuterol and pork mass. Three pork samples were prepared for each concentration.

The pre-processing steps of samples before detection are shown in Figure 1. First, 2 mL of 10.0% sodium carbonate solution and 6 mL of pure ethyl acetate solvent were added to a centrifuge tube, and the mixture was vortexed and mixed for 1 min. The mixture was centrifuged at a speed of 10,000 r/min for 5 min after full extraction. Later, the upper organic solvent was sucked into a centrifuge tube, and heated by a water bath, and condensed until it nearly dried. Finally, 2 mL of ultrapure water was added to a conical flask to redissolve the residue. Clenbuterol solution redissolved was stored in a 4 °C environment for testing.

### 2.5. Spectral Collection

As shown in Figure 1, when collecting the spectra of samples, the mixed solution of 2.5 µL gold colloid, 2.5 µL clenbuterol aqueous solution, and 1.5 µL NaCl solution were dropped onto an aluminum substrate for detection. The gold nanoparticles can be aggregated by adding NaCl solution. The same three samples were chosen for spectral collection under each concentration gradient, and three Raman spectra were collected each sample. 

### 2.6. Data Processing

Affected by various factors such as system fluctuations, changes in the external environment, and the fluorescence background generated by impurities, the obtained spectra signals contained other irrelevant information and fluorescence interference in addition to the information of the target substance. Therefore, the fluorescence background of the Raman spectra is removed using automatic Whitacre fitting algorithm (AWF), and multiple scattering correction (MSC) was used to eliminate baseline drift. The original and pretreated SERS spectra of clenbuterol in pork are shown in Figure S1. The performance of the models was evaluated according to the correlation coefficient (*R*^2^) and root mean square error of correction (*RMSEC*) [23]. If the correlation coefficient is high and the root mean square error is low, it indicates that the established model has a better performance [24]. MATLAB 2016a was used to process the data.

## 3. Results and Discussion

### 3.1. Characterization of the Gold Colloids

**Figure 2:**
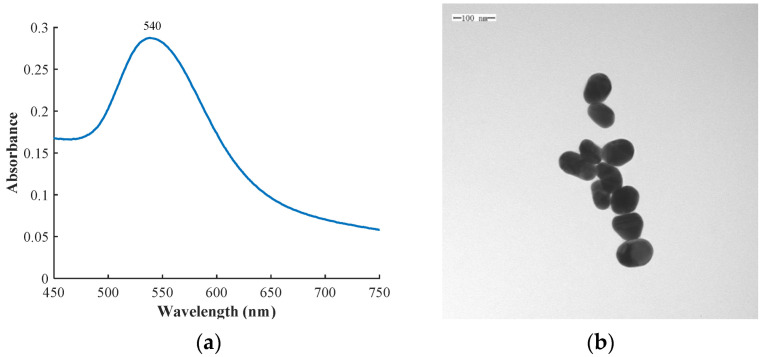
(**a**) UV–vis absorption spectra of gold colloid; (**b**) TEM image of gold colloid.

UV/vis absorption spectra were used to characterize the optical properties of the colloidal solution, shown in Figure 2a. The peak position and width can be used to estimate the particle size and dispersity of gold colloid. A redshift and broadening occur with increasing particle size, followed by an increase in size and polydispersity [25]. A TEM image of the gold colloid is shown in Figure 2b, and it can be seen that the sizes and shapes of the gold colloid were uniform.

### 3.2. SERS Spectra of Clenbuterol in Different Solvents

**Figure 3:**
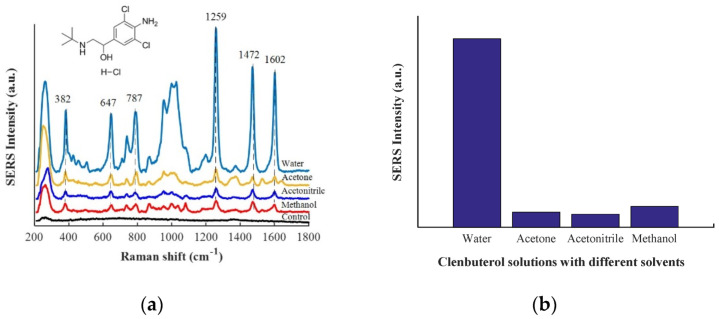
(**a**) SERS spectra of clenbuterol (10 µg/mL) in different solvents and SERS spectra of water as a control group; (**b**) SERS intensity of clenbuterol at 1259 cm^−1^ in different solvents.

In this report, SERS spectra of clenbuterol (10 µg/mL) in four different solvents of water, acetonitrile, acetone and methanol were compared. From Figure 3a, it can be seen that SERS spectra of clenbuterol in different solvents had similar characteristic bands. SERS spectra of clenbuterol aqueous solution and water blank control group were compared, and it was found that the bands at 382, 647, 787, 1259, 1472, and 1602 cm^−1^ were attributed to clenbuterol. The in-plane deformation of the phenyl ring coupled to C-C-Cl deformations band is at 382 cm^−1^; the band at 647 cm^-1^ is attributed to out-of-plane ring deformation; the band at 787 cm^−1^ can be assigned to stretching vibration of C-Cl coupled to in-plane stretching ring; the band at 1259 cm^-1^ is linked to the stretching of C-N coupled to in-plane ring deformations; the band centered at 1472 cm^−1^ is assigned to the in-plane stretching vibrations coupled with the anilines C-N stretching vibration [26]; and the band at 1602 cm^−1^ can be assigned to the C = C stretching band of the aromatic ring [27]. Figure 3b shows the SERS intensity of clenbuterol at 1259 cm^−1^ in different solvents, from which we can see that clenbuterol has the best enhancement performance in aqueous solution. This may be due to clenbuterol in organic solvent being difficult to bind to gold colloid due to the competitive adsorption of organic solvent molecules. Subsequently, ultrapure water was used to redissolve the extracted clenbuterol.

### 3.3. Optimization of Sample Clean-Up

**Figure 4:**
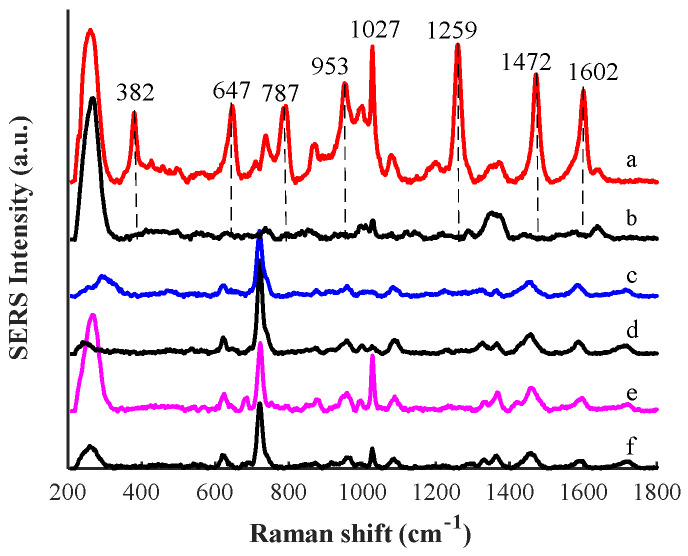
SERS spectra of clenbuterol (10 µg/g) in pork with different extractants: curves a, c, and e represent the SERS spectra of clenbuterol in pork when ethyl acetate, acetonitrile, and methanol were used as extraction agents, respectively; curves b, d, and f represent the control group of SERS spectra of pork when ethyl acetate, acetonitrile, and methanol were used as extraction agents, re-spectively.

CB is a weakly alkaline compound (pKa = 9.6). When pH > pKa, CB is soluble in organic solvents. Otherwise, the CB is soluble in water when pH < pKa [28]. When ethyl acetate and sodium carbonate solution were used as the extraction agent, CB dissolved into ethyl acetate because sodium carbonate solution was alkaline. In addition, ethyl acetate is not mutually soluble with water and can be separated by centrifugation. Likewise, acetonitrile and methanol were compared as extractants. For extraction, 1 mL of saturated sodium chloride solution and 6 mL of acetonitrile or methanol were added to a centrifuge tube containing clenbuterol pork samples of 10 µg/g. Saturated sodium chloride solution can reduce the solubility of CB in water and stratify with acetonitrile. Therefore, acetonitrile can be separated with water by centrifugation. Since methanol and water are mutually soluble in infinite proportions, an additional 1 g of anhydrous Na_2_SO_4_ was added to absorb excess moisture when methanol was used as an extractant. Since clenbuterol has the best enhancement in aqueous solution, ultrapure water was chosen to redissolve the residue of the concentrated extract. As shown in Figure 4, curves a, c, and e represented the SERS spectra of clenbuterol in pork when ethyl acetate, acetonitrile, and methanol were used as extraction agents, respectively. Curves b, d, and f represent the control group of SERS spectra of pork when ethyl acetate, acetonitrile, and methanol were used as extraction agents, respectively. It can be seen from Figure 4 that ethyl acetate extraction had a better enhancement performance, and the bands at 382, 647, 787, 953, 1259, 1472, and 1602 cm^−1^ of clenbuterol can be observed, which have the same bands as the clenbuterol aqueous solution. The band at 1027 cm^−1^ observed on curves a and b was caused by fat in pork. As can be seen from curves c, d, e, and f, when acetonitrile and methanol were used as extraction agents, the SERS spectra of clenbuterol in the pork had similar bands to the control group, so they could not be used to determine whether it contained clenbuterol or not. Therefore, ethyl acetate has a better extraction effect on clenbuterol from pork.

### 3.4. Effect of Different Concentrations of Aggregating Compounds on Enhancement Raman Spectra

The enhancement performance will be influenced by the charge properties and binding degree between metal nanoparticles and different molecules. The charge balance is upset when electrolytes are added, which causes particles to coalesce [29]. The concentration of NaCl can influence the degree of aggregation of AuNPs [30,31]. Due to the highly localized nature and extremely large signal enhancement of SERS hot spots, it is believed that a small fraction of analyte molecules adsorbed into hot spots is responsible for the majority of the total SERS signal [32]. Therefore, an aggregating compound is routinely added to the substrate to achieve a degree of enhancement of SERS signals [33].

**Figure 5:**
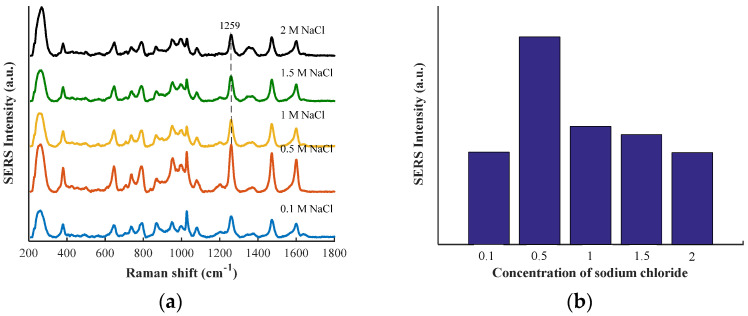
(**a**) SERS spectra of clenbuterol in pork using NaCl solutions at different concentrations as aggregating compounds; (**b**) SERS intensity of clenbuterol in pork at 1259 cm^−1^ using different con-centrations of NaCl solution as aggregating compounds.

As shown in Figure 5a, SERS spectra of clenbuterol in pork all had the same bands and different peak intensities using sodium chloride with different concentrations as aggregating compounds. The SERS intensities of clenbuterol in pork at 1259 cm^−1^ using NaCl solution with different concentrations as aggregating compounds are shown in Figure 5b. It can be observed that with the increase of NaCl concentration, SERS intensity of clenbuterol in pork at 1259 cm^−1^ first increased and then decreased, which may be due to the gradual aggregation of uniformly dispersed gold nanoparticles. Since clenbuterol is attached to the gold nanoparticles, when the gold nanoparticles gradually gather, the quantity of the target substance at the “hot spot” gradually increases. With the aggregation of gold nanoparticles, agglomeration precipitation will occur, which is not conducive to the enhancement of Raman signals of target molecules. As can be seen from Figure 5b, the enhancement effect of clenbuterol in pork was best when using 0.5 M of sodium chloride as the aggregating compound.

### 3.5. Repeatability of Experiment

**Figure 6:**
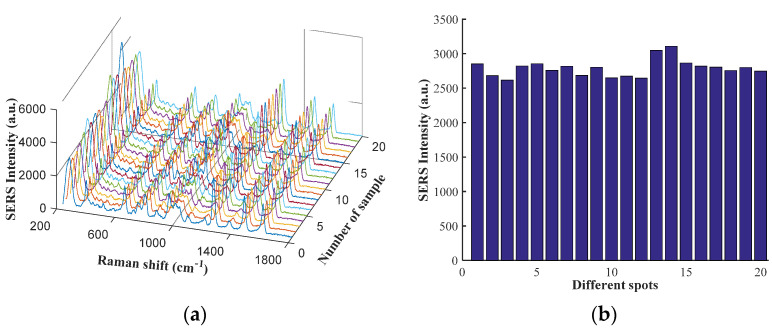
(**a**) SERS spectra of pork containing clenbuterol (10 µg/g); (**b**) SERS intensities of clenbuterol in pork at 1259 cm^−1^.

A total of 20 samples of pork contenting clenbuterol (10 µg/g) were selected to verify the repeatability of the proposed method. SERS spectra of pork containing clenbuterol (10 µg/g) are shown in Figure 6a. SERS intensities of samples at 1259 cm^−1^ are shown in Figure 6b. It can be seen that the signal has a better consistency. The relative standard deviations of SERS intensities at 382, 647, 787, 1259, 1472, and 1602 cm^−1^ were 6.9%, 6.2%, 6.0%, 4.6%, 5.1%, and 5.0%, respectively. The results show that this method has a better repeatability and stability.

### 3.6. Quantitative Analysis

Pork samples with different clenbuterol content (1, 3, 5, 7, 9, and 10 µg/g) were prepared for quantitative analysis. Ethyl acetate and 10% sodium carbonate solutions were selected as extractants, and the pork samples were treated using the pre-treatment method described in Section 2.4. The SERS spectra were collected using gold colloid as the enhanced substrate and 0.5 M of NaCl solution as the aggregating compound.

**Figure 7:**
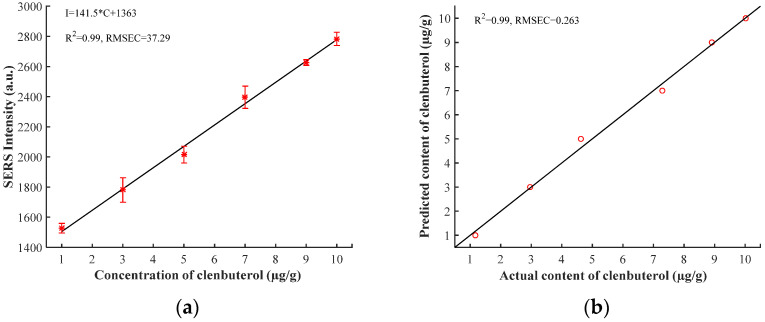
(**a**) The relationship between Raman intensity and clenbuterol content of samples. (**b**) The plot of the clenbuterol content of samples determined by SERS versus the actual clenbuterol content of samples.

Since the original spectra collected had different fluorescence background signals, MSC was used to eliminate baseline drift, and AWF was used to remove the baseline. The characteristic band intensity of clenbuterol at 1472 cm^−1^ was selected for data analysis. As is shown in Figure 7a, a better linear relationship between the clenbuterol content of samples and SERS intensity was found. The linear equation between clenbuterol content of samples and SERS intensity at 1472 cm^−1^ is as follows:

I = 141.5 × C + 1363(1)

where C (µg/g) is the clenbuterol content of samples and I is the SERS intensity of clenbuterol at 1472 cm^−1^.

The peak intensities at 1472 cm^−1^ of spectra were interpolated into the linear equation to verify the prediction performance of the model. As is shown in Figure 7b, the model had a better performance with good linearity (*R*^2^ = 0.99) and lower root mean square error (*RMSEC* = 0.263 µg/g).

## 4. Discussion

In this report, SERS technology was used to detect clenbuterol in pork. Compared with the traditional detection method, this method has the advantages of simple operation, short detection time and fast detection speed, which can be used for the crude screening of pork containing clenbuterol in the market. However, the detection limit is 1 µg/g, which is relatively high. A better enhancement substrate is needed to reduce the detection limit in the future. In addition, with the rapid hardware update of Raman system and the gradual deepening of stoichiometry, the detection limit will be gradually reduced.

## 5. Conclusions

In this report, different extraction agents were compared, and it was found that clenbuterol in pork had a better enhancement effect using ethyl acetate as an extraction agent. Furthermore, the clenbuterol signals in several different solvents were compared, and it was found that clenbuterol had a better enhancement effect in aqueous solution. Therefore, water was chosen as the solvent for clenbuterol detection. Then, NaCl with different concentrations as aggregating compounds were compared, and the results showed the enhancement effect was best using 0.5 M NaCl as the aggregating compound. Finally, pork samples with different clenbuterol content (1, 3, 5, 7, 9, and 10 µg/g) were prepared for quantitative analysis. The SERS spectra of samples were collected with 0.5 M of NaCl solution as the aggregating compound and gold colloid as an enhanced substrate. MSC and AWF were used to eliminate baseline drift and remove the fluorescence background contained in the original Raman spectra, respectively. A unary linear regression model was established between SERS intensity at 1472 cm^−1^ and clenbuterol content in pork samples. The established model had a better linear relationship, with a correlation coefficient *R*^2^ of 0.99 and a root mean square error of 0.263 µg/g. This method can be used for rapid crude screening of pork containing clenbuterol in the market.

## Data Availability

Not applicable.

## Supplementary Materials

The following are available online at https://www.mdpi.com/article/10.3390/bios12100859/s1, Figure S1: (a) Original SERS spectrum of pork containing clenbuterol; (b) SERS spectrum after pretreatment of pork containing clenbuterol.
