# Supplementary material for: Rapid Detection of Clenbuterol Residues in Pork Using Enhanced Raman Spectroscopy
Source: Biosensors (Basel). 2022 Oct 11;12(10):859. doi: 10.3390/bios12100859 (PMC9599483; doi:10.3390/bios12100859)
Supplement: Supplementary file 1 [file biosensors-12-00859-s001.zip › biosensors-1929580-supplementary.pdf]

Supplementary material

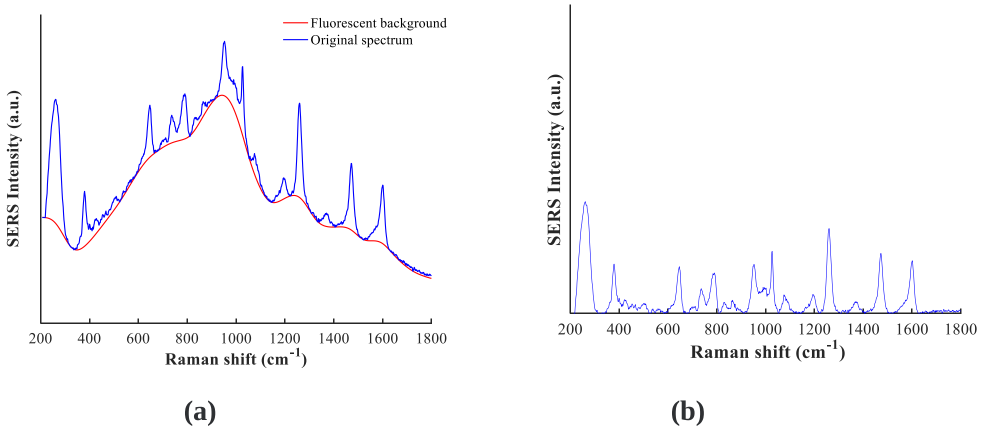

Figure S1. (a) Original SERS spectrum of pork containing clenbuterol; (b) SERS spectrum after pretreatment of pork containing clenbuterol.
